# A flow cytometry approach reveals heterogeneity in conventional subsets of murine renal mononuclear phagocytes

**DOI:** 10.1038/s41598-021-92784-x

**Published:** 2021-06-24

**Authors:** Johannes Nordlohne, Ilona Hulsmann, Svenja Schwafertz, Jasmin Zgrajek, Manuel Grundmann, Sibylle von Vietinghoff, Frank Eitner, Michael S. Becker

**Affiliations:** 1 Cardiovascular Research, Research and Development, Pharmaceuticals, Kidney Diseases, Bayer AG, Building 0500, 214, 42113 Wuppertal, Germany; 2 Nephrology Section, Medical Clinic 1, University Hospital Bonn, Rheinische Friedrich-Wilhelms University, Bonn, Germany

**Keywords:** Kidney diseases, Monocytes and macrophages

## Abstract

Mononuclear phagocytes (MNPs) participate in inflammation and repair after kidney injury, reflecting their complex nature. Dissection into refined functional subunits has been challenging and would benefit understanding of renal pathologies. Flow cytometric approaches are limited to classifications of either different MNP subsets or functional state. We sought to combine these two dimensions in one protocol that considers functional heterogeneity in each MNP subset. We identified five distinct renal MNP subsets based on a previously described strategy. In vitro polarization of bone marrow-derived macrophages (BMDM) into M1- and M2-like cells suggested functional distinction of CD86 + MHCII + CD206- and CD206 + cells. Combination of both distinction methods identified CD86 + MHCII + CD206- and CD206 + cells in all five MNP subsets, revealing their heterologous nature. Our approach revealed that MNP composition and their functional segmentation varied between different mouse models of kidney injury and, moreover, was dynamically regulated in a time-dependent manner. CD206 + cells from three analyzed MNP subsets had a higher ex vivo phagocytic capacity than CD86 + MHCII + CD206- counterparts, indicating functional uniqueness of each subset. In conclusion, our novel flow cytometric approach refines insights into renal MNP heterogeneity and therefore could benefit mechanistic understanding of renal pathology.

## Introduction

Acute and chronic forms of kidney injury constitute a major health concern as they are associated with an increased mortality rate^1–3^. Cells of the mononuclear phagocyte (MNP) system, including monocytes, dendritic cells (DC) and macrophages, are on one side major contributors in disease progression after kidney injury by driving inflammation but simultaneously mount tissue repair and resolution of inflammation^4–7^. This functional diversity is reflected in a wide range of phenotypical characteristics and has made the identification of functional subunits in this complex MNP network challenging^8–10^.

In order to characterize distinct MNP subsets in the kidney, flow cytometric approaches have utilized surface markers CD11b, F4/80, Ly6C, and/or CD11c for distinction of at least three^11–16^ or even up to five unique subsets^17,18^. Drawing conclusions about the mechanistic relevance of these MNP subsets in disease models warrants careful consideration as one cannot necessarily imply their functional uniformity. Indeed, single-cell RNA sequencing revealed multimodal expression of pro- and anti-inflammatory genes among individual MNP subsets^13,19^, indicating additional layers of complexity in these subsets. This level of heterogeneity extends also into mechanistical studies, as the same F4/80^high^ MNP subset has been implicated both in progression from acute to chronic kidney injury^20^ but also in recovery from acute kidney injury^15^. Results from depletion experiments affecting whole MNP subsets via clodronate liposomes or promotor-specific diphteria toxin receptor have also been rather inconclusive so far^21–24^, raising the need for a more granular analysis.

Kidney MNPs are often categorized into a pro-inflammatory or wound-healing cluster in order to characterize their role after kidney injury. This functional dichotomy is for example reflected in the M1/M2 paradigm, which comprises a M1 component with pro-inflammatory cytokine and chemokine secretion and a M2 component with immune-regulatory, wound healing and fibrotic properties^25–27^. In this context, CD86 and MHCII expressing cells have been associated with histological and functional injury, while CD206 expressing cells are associated with fibrotic and reparative processes^28–30^. Such binary distinctions have been used frequently to determine the overall inflammatory state of renal MNPs but often on preselected subsets or without consideration of different MNP subsets.

In order to surmount the limitations in granularity of the above ascribed methods, we aimed to establish an easily accessible flow cytometric method that combines MNP subset distinction and surface marker-based functional distinction in order to comprehensively understand renal MNP complexity Furthermore, we aimed to employ this newly established method to characterize MNP subsets in several preclinical kidney injury models which are heavily associated with MNP infiltrates.

## Results

### Five renal MNP subsets are defined by distinct surface marker expression and accumulate after kidney injury

**Figure 1:**
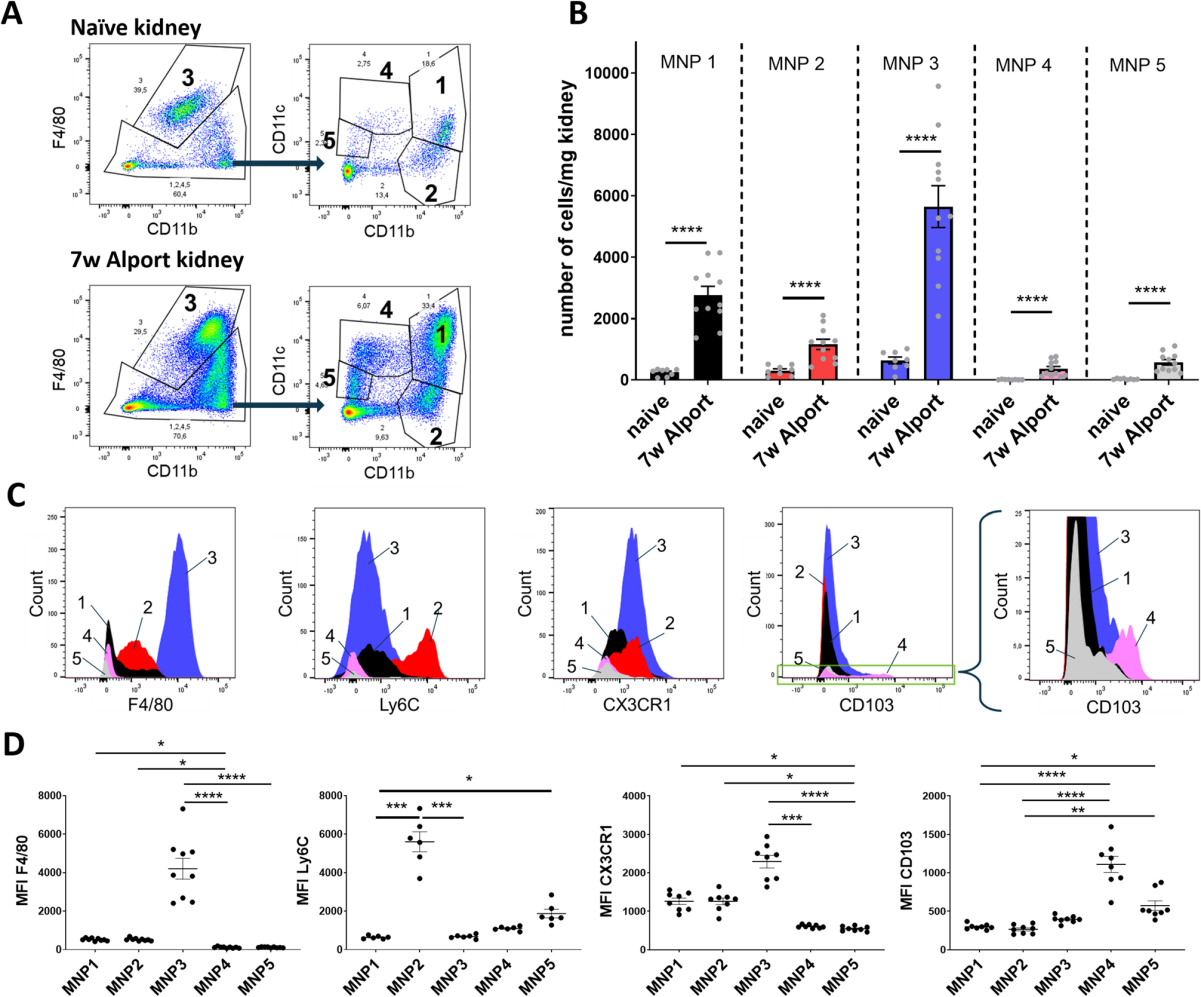
Markers F4/80, CD11b, and CD11c distinguish five distinct MNP subsets in the murine kidney. (**A**) A gating strategy for five renal MNP subsets was adopted from Kawakami et al. and representative FACS plots are shown of a naïve mouse and 7 weeks old *Col4a3*^*−/−*^ mice with Alport syndrome as an example for diseased state. (**B**) Quantification of cell numbers in the five MNP subsets for naïve (n = 8) and *Col4a3*^*−/−*^ mice (n = 11). Shown are pooled data from two independent experiments. y = ln(y) transformed cell numbers were compared by unpaired t-test *P < 0.05, **P < 0.01, ***P < 0.001, ****P < 0.0001. (**C**) Representative histograms for expression of surface markers F4/80, Ly6C, CX3CR1 and C103 in all five MNP subsets. (**D**) Quantification of geometric mean fluorescence intensity (MFI) of surface markers. MFI were compared by Kruskal–Wallis with post-hoc Dunn’s multiple comparisons test *P < 0.05, **P < 0.01, ***P < 0.001, ****P < 0.0001.

Dissection of the multi-facetted nature of MNPs has been challenging and often been restricted to either phenotypical or functional distinction via flow cytometry, which we sought to combine. For the first part of our flow cytometric method we adopted a phenotypical characterization method for renal MNPs from Kawakami et al.^17^ because it successfully segregates five MNP subsets with the use of only few surface markers. Following this strategy, we segregated five unique MNP subsets in murine kidneys with the surface markers F4/80, CD11b and CD11c (Fig. 1A). These markers are commonly used among others like Ly6C or CX3CR1 to differentiate MNP subsets in the kidney. In line with Kawakami et al., in naïve kidneys, kidney resident F4/80^high^ macrophages (MNP subset 3) and CD11b^high^ MNPs (subsets 1 and 2) were more abundant than DC-like CD11b^medium^CD11c^high^ (subset 4) and CD11b^low^CD11c^medium^ (subset 5) cells (Fig. 1A,B). To our knowledge the method by Kawakami et al. has not been used in physiological models of kidney injury so far. To test how MNP subset dynamics may be influenced by kidney injury we therefore analyzed kidney MNPs isolated from *Col4a3*^*−/−*^ mice with Alport syndrome (Fig. 1A,B). While MNP subsets 1, 2 and 3 were already detected in naïve murine kidneys in relatively large numbers, subsets 4 and 5 became clearly apparent in kidneys from *Col4a3*^*−/−*^ mice (Fig. 1B). We confirmed the uniqueness of these five subsets by assessing the expression of other distinct surface markers on these cells (Fig. 1C,D): By nature of our gating strategy, subset 3 had the highest expression of the classical macrophage marker F4/80, which also displayed intermediate expression on parts of subset 2. Expression of the inflammatory monocyte marker Ly6C was restricted to subset 2. Subsets 1, 2 and 3 had also notable expression of the chemokine receptor CX3CR1. CD103 is an integrin that can be found on conventional type 1 DCs (cDC1) and was restricted to subset 4. Fluorescence minus one (FMO) controls are available in Supplementary Figure S1. These data demonstrate that in line with the strategy by Kawakami et al. we were able to distinguish five distinct renal MNP subsets with unique surface marker expression. Moreover, all five MNP subsets were dynamically increased in diseased kidneys from *Col4a3*^*−/−*^ mice.

### CD206, CD86 and MHCII expression differentiate functionally distinct subsets

**Figure 2:**
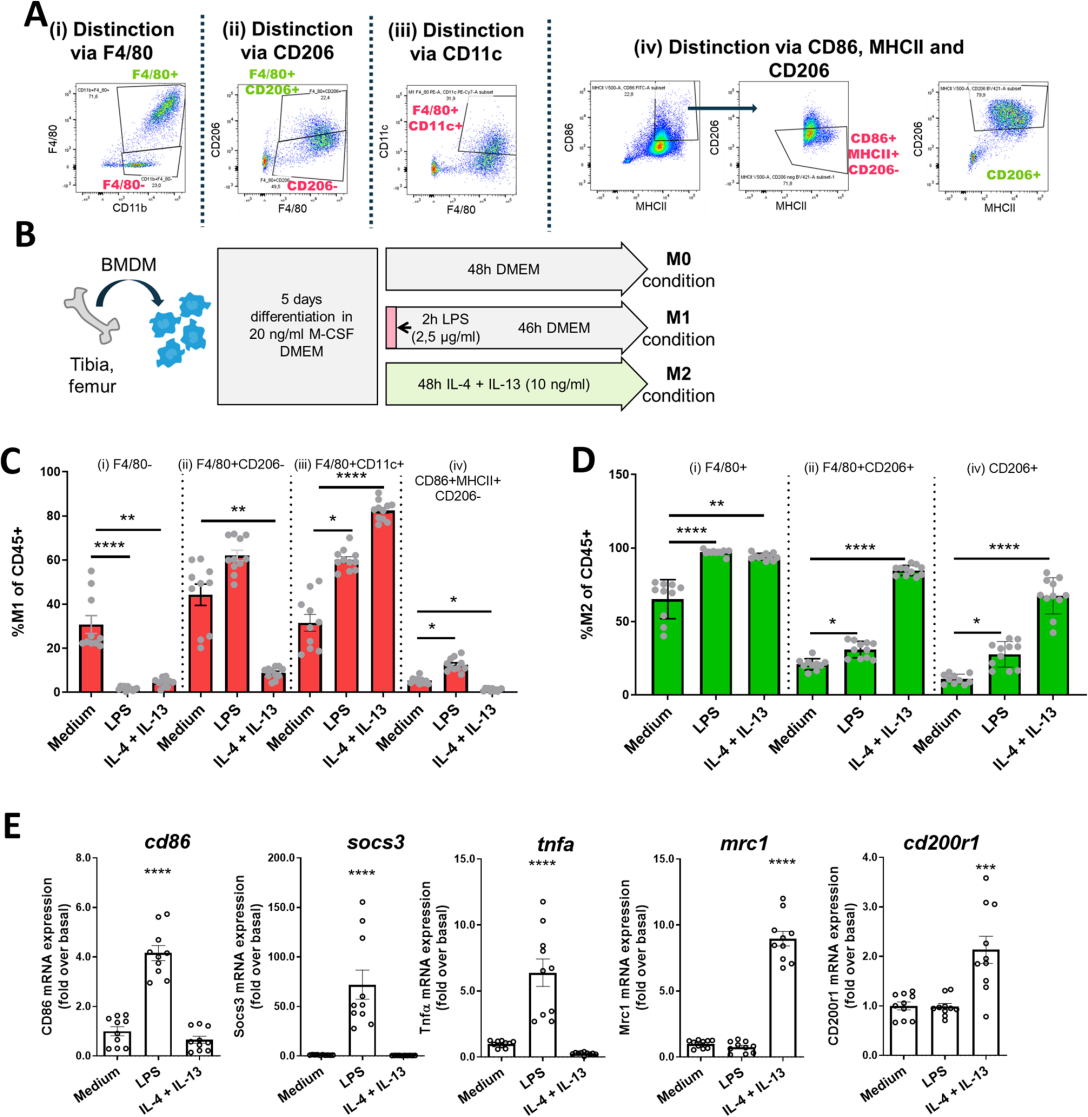
CD86, MHCII, and CD206 identify M1- and M2-like cells in in vitro stimulated BMDM. (**A**) We applied gating strategies for M1/M2 that are commonly used in literature (i–iii) and an in-house approach (iv) to in vitro stimulated BMDM. (**B**) For in vitro polarization BMDM were differentiated for 5 days with M-CSF and then stimulated with LPS or IL-4 and IL-13. After 48 h cells were analyzed by flow cytometry and M1- (**C**) and M2-like cells (**D**) were quantified with each strategy from (**A**). (**E**) Duplicates were subjected to qPCR to determine expression of M1 (cd86, socs3, tnfa) and M2 (mrc1, cd200r1) genes. (n = 10 from 3 independent experiments). Kruskal–Wallis with post-hoc Dunn’s multiple comparisons test (**C** and **D**) or one way ANOVA with post-hoc Dunnett's (**E**) multiple comparisons test against medium *P < 0.05, **P < 0.01, ***P < 0.001, ****P < 0.0001.

To further separate the five renal MNP subsets into functional subunits we sought to utilize surface markers that have been associated with functional distinctiveness before. In order to identify suitable markers for this, we referred to markers previously used to characterize in vitro polarized M1- and M2-like cells, as they represent two functionally distinct entities^31,32^. We compared several flow cytometric approaches that have been employed in murine kidneys in order to differentiate cells, that are reminiscent of M1- and M2-like cells (Fig. 2A): (i) M1-like: F4/80^low^ cells; M2-like: F4/80^high^ cells^33,34^, (ii) M1-like: CD206^low^ cells; M2-like: F4/80^high^ and CD206^high^ cells^35,36^, (iii) and M1-like: F4/80^high^ and CD11c^high^ cells^37^. Additionally, we employed a fourth strategy (iv): M1-like:CD86^high^ and MHCII^high^ and CD206^low^; M2-like: CD206^high^ (FMO and isotype controls available in Supplementary Figure S2). Bone marrow-derived macrophages (BMDM) were stimulated with LPS or IL-4 and IL-13 to induce M1- or M2- like phenotypes, respectively (Fig. 2B). Gating strategies (ii) and (iv) were qualified best to detect the increase in M1-like cells after LPS stimulation (Fig. 2C). The additional criterium for CD86^high^ cells of M1-like cells in gating strategy (iv) resulted in a more stringent selection than gating strategy (ii) hence fewer cells. M2-like cells after IL-4/IL-13-stimulation were detected by CD206 in gating strategies (ii) and (iv) (Fig. 2D) and the addition of marker F4/80 in (ii) did not change cell numbers compared to (iv) as almost all CD206^high^ cells were also F4/80^high^ after in vitro culture. Because we were also interested in quantifying cells of MNP subsets 1, 2, 4, and 5, which all have lower F4/80 expression, we adopted gating strategy (iv) for our in vivo analyses. RNA analysis of common M1 and M2 genes confirmed successful polarization of BMDMs (Fig. 2E). These data demonstrate that gating strategy (iv) was well suited to differentiate two functionally distinct subsets (CD86 + MHCII + CD206- and CD206 +) in vitro.

### Combination of gating for MNP subsets and functional subsets results in technical robust identification of novel subsets

**Figure 3:**
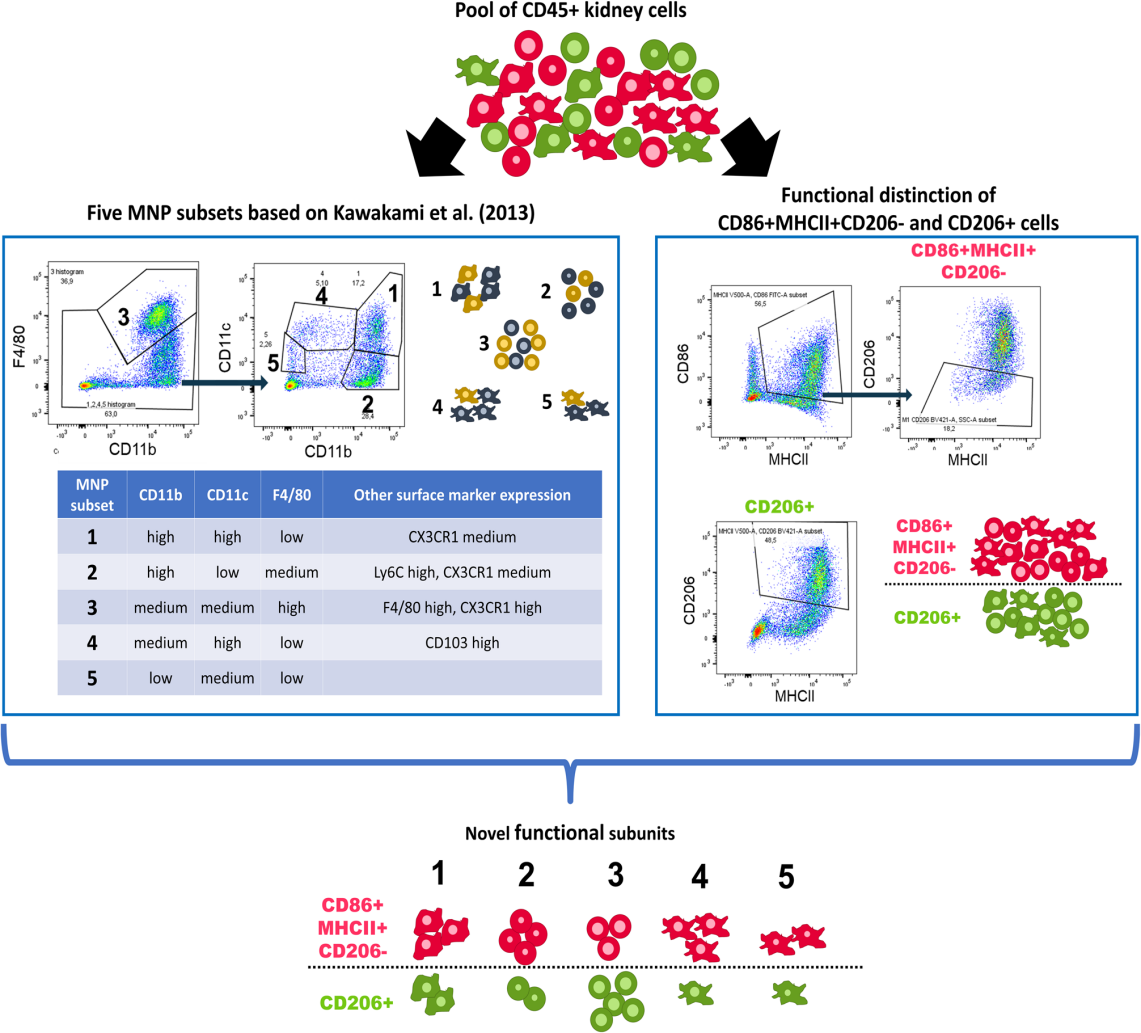
Combination of phenotypical MNP subset analysis and functional distinction grants additional dimension to flow cytometric analysis. Flow cytometry of renal leukocytes (pre-gated on CD45 + Ly6G-, different cells are depicted schematically) is often either used to distinguish MNP subsets with specific surface markers (left box) or functionally distinct cells (right box). We propose a combined analysis of both dimensions for comprehensive understanding of functional and phenotypical state on a single cell level.

Next, we employed our gating for CD86 + MHCII + CD206- and CD206 + subsets on top of the gating strategy for the five MNP subsets in order to gain an impression on the functional heterogeneity of the five MNP subsets (Supplementary Figure S3). With this technique we extend the one-dimensional way of assessing either the MNP subset or the functional state into an analysis that gives both information on a single-cell level (Fig. 3).

We investigated the technical variance of processing the cells for flow cytometry by evenly distributing a digested kidney sample between different experimenters and subjecting triplicates to our washing, staining and analysis protocol. The results suggest a very low technical variance between different experimenters and their replicates when considering total counts of leukocytes, individual MNP subsets and CD86 + MHCII + CD206- and CD206 + cells (Supplementary Figure S4). These results confirm a high technical robustness of our flow cytometry method.

### MNP subsets display functional heterogeneity and are regulated in a model-specific manner

**Figure 4:**
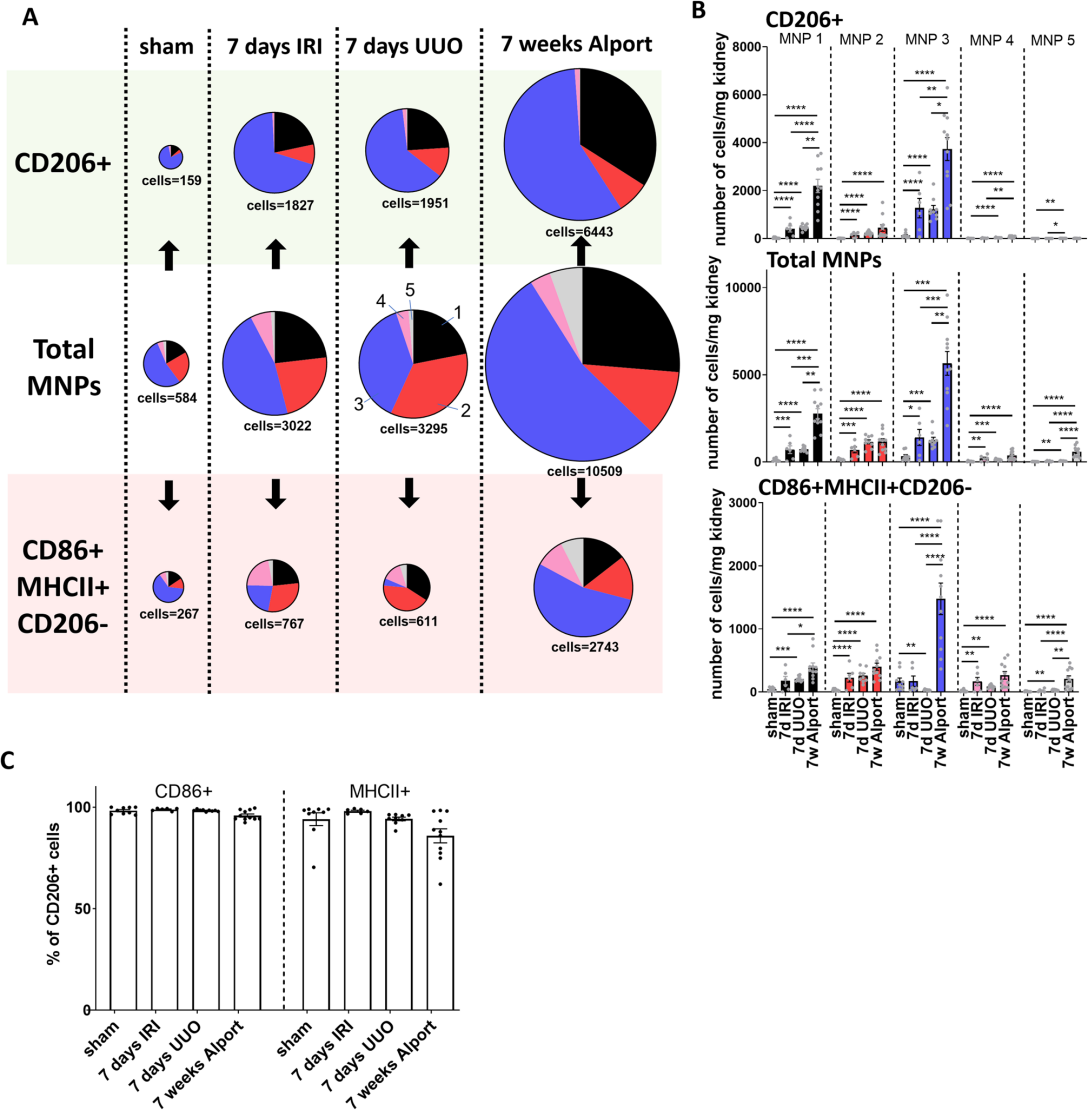
Different models of kidney injury possess a fingerprint-like MNP subset composition collectively but also in CD206 + and CD86 + MHCII + CD206- cells. (**A**) MNP subsets were quantified among total leukocytes (mid row) in different models of kidney injury (means of n = 9 for 7 days UUO sham control, n = 6 for IRI, n = 9 for UUO, n = 11 for Alport; cell number is given in cells per mg kidney). Our approach additionally allowed the quantification of functionally distinct CD206 + (top row) and CD86 + MHCII + CD206- (bottom row) cells in each MNP subset. (**B**) Quantification of cell numbers from (**A**) as bar graph. (**C**) Percent CD86 + and MHCII + cells among CD206 + cells. One-way ANOVA followed by Tukey’s multiple comparisons test of y = ln(y) transformed data *P < 0.05, **P < 0.01, ***P < 0.001, ****P < 0.0001.

In a first in vivo application of our newly established method we tested if there are differences in MNP subset composition between three commonly used models of kidney injury: 7 days of unilateral ureter obstruction (UUO), ischemia reperfusion injury (IRI) after 7 days and 7 weeks old *Col4a3*^*−/−*^ mice with Alport syndrome (Fig. 4A,B). While the total amount of MNPs and CD86 + MHCII + CD206- and CD206 + cells were comparable between UUO and IRI (Fig. 4A, size of pie charts, statistics are depicted in Supplementary Figure S5 A and B), the additional MNP subset analysis granted by our technique revealed relative changes in MNP composition (Fig. 4A, Supplementary Figure S5 A and B). There was a relative increase in MNP subset 1 and a relative decrease in MNP subset 3 among CD86 + MHCII + CD206- cells in the UUO compared to the IRI kidneys. Composition of the CD206 + cells was in both models comparable with strongest contribution from subset 3 followed by subset 1. Kidneys from 7 weeks old *Col4a3*^*−/−*^ mice with Alport syndrome contained strikingly more cells in total than the other two models (Fig. 4B) except in MNP subset 2 and 4. Especially CD86 + MHCII + CD206- cells in MNP subset 3 were increased while subset 1 contributed more to the pool of CD206 + cells. All three models displayed a robust increase in virtually all MNP subsets compared to “healthy” sham control kidneys from UUO (Fig. 4B), while changes between control kidneys from all three models were minor (Supplementary Figure S5C). Of note, almost all CD206 + cells exhibited CD86 and MHCII expression in vivo (Fig. 4C and Supplementary Figure S3B). In summary, these data show that all MNP subsets may harbor cells of different functional states after kidney injury and further demonstrate that the composition of kidney MNPs and their inflammatory state is highly dependent on the context of tissue injury.

### Dynamic regulation of different MNP subsets is highly dependent on the model of kidney injury

**Figure 5:**
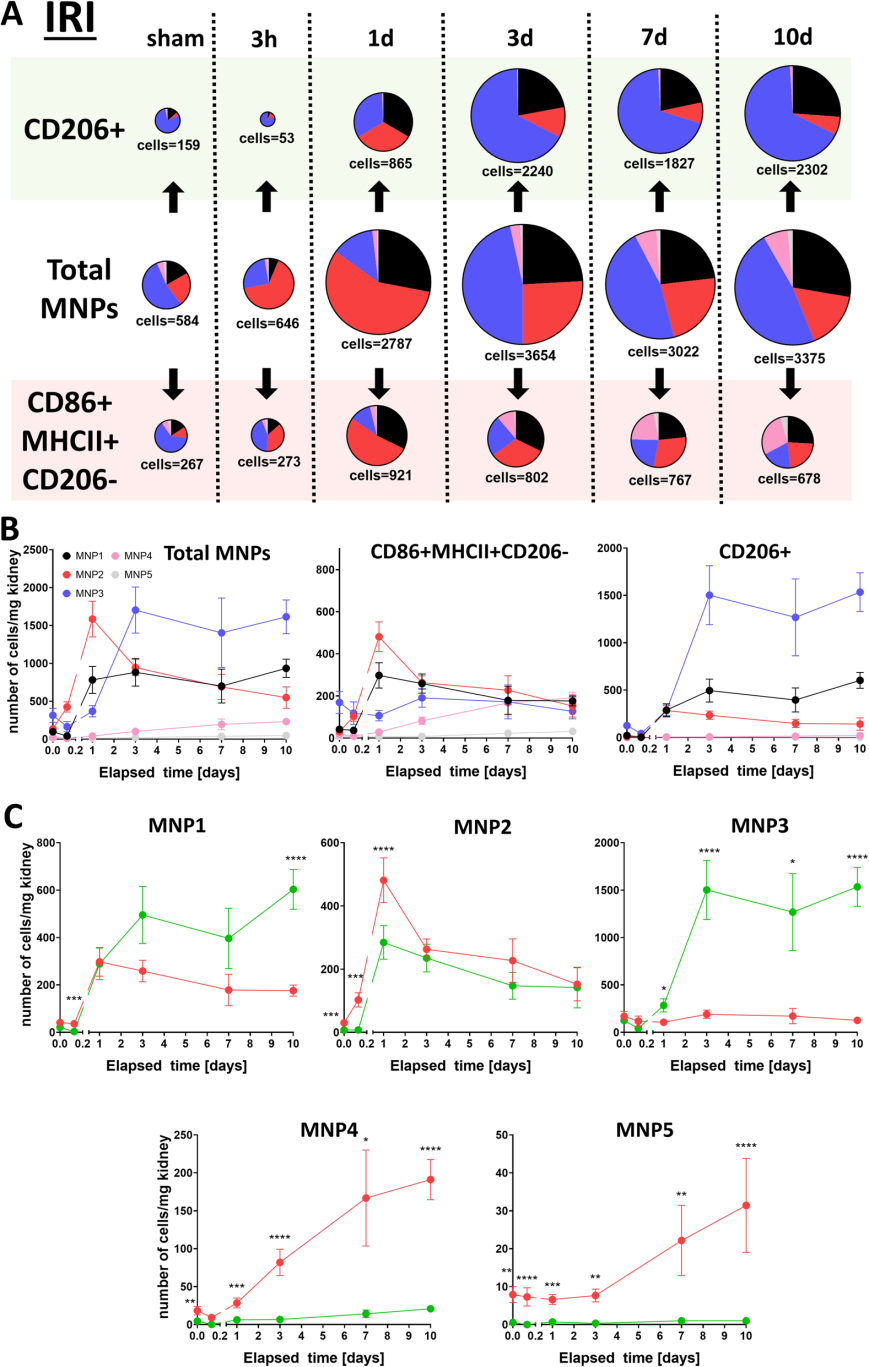
CD206 + and CD86 + MHCII + CD206- cells in MNP subsets are dynamically regulated after IRI. MNP composition was monitored over a 10 day time course in a kidney injury model of IRI (n = 9 for sham, n = 10 for 3 h, n = 10 for 1d, n = 12 for 3d, n = 6 for 7d, n = 7 for 10d): Data are depicted as pie charts with their size corresponding to the amount of total cells (**A**, cell number is given as mean in cells per mg kidney) or plotted on a time axis grouped into CD206 + and CD86 + MHCII + CD206- cells (**B**) or MNP subsets (**C**, red = CD86 + MHCII + CD206-, green = CD206 +). Cell numbers for each time point in (**C**) were compared by unpaired t test of y = ln(y) transformed data *P < 0.05, **P < 0.01, ***P < 0.001, ****P < 0.0001.

**Figure 6:**
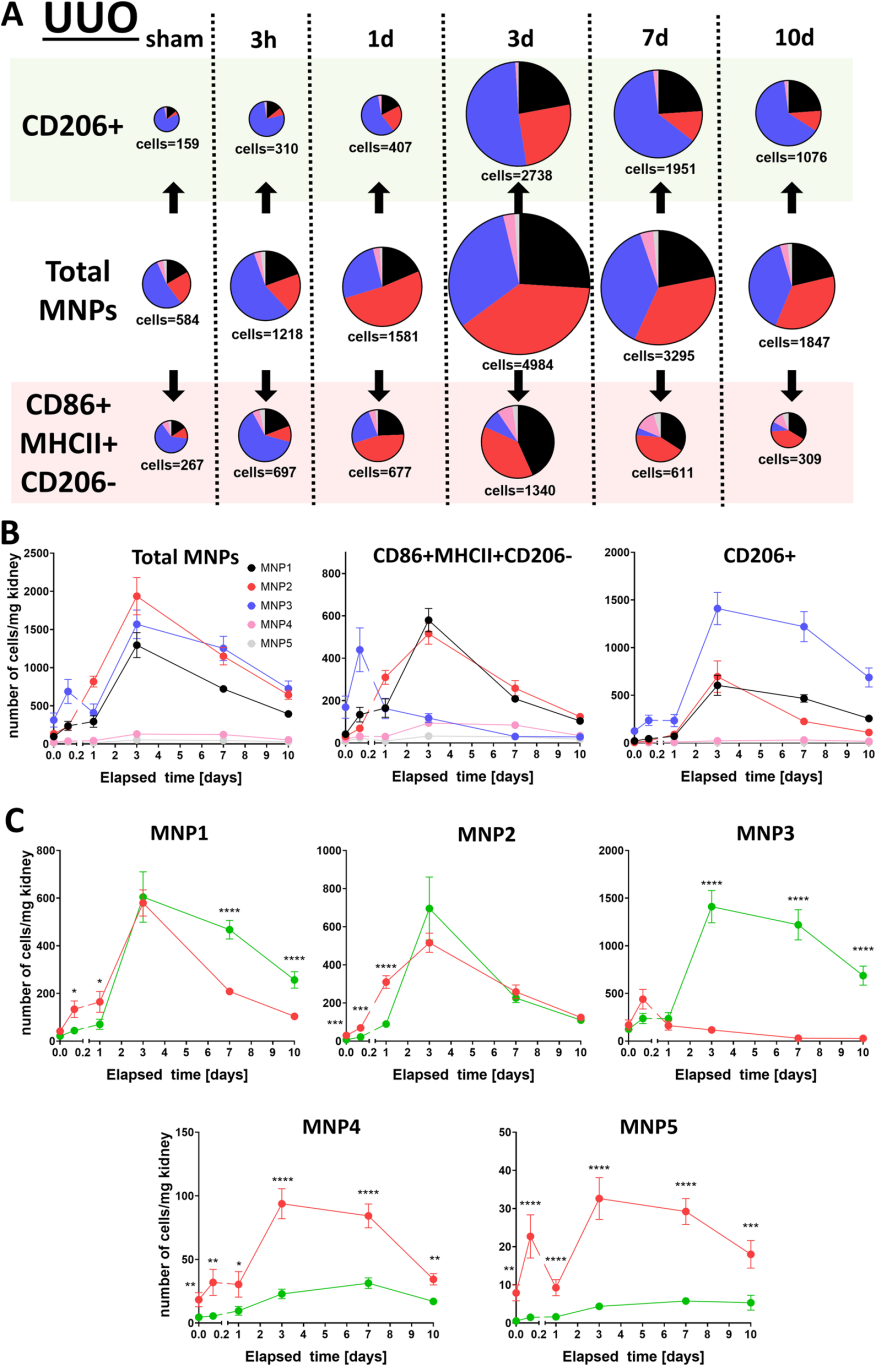
CD206 + and CD86 + MHCII + CD206- cells in MNP subsets are dynamically regulated after UUO. MNP composition was monitored over a 10 day time course in a kidney injury model of UUO (n = 6 for sham, n = 10 for 3 h, n = 8 for 1d, n = 8 for 3d, n = 9 for 7d, n = 9 for 10d): Data are depicted as pie charts with their size corresponding to the amount of total cells (**A**, cell number is given as mean in cells per mg kidney) or plotted on a time axis grouped into CD206 + and CD86 + MHCII + CD206- cells (**B**) or MNP subsets (**C**, red = CD86 + MHCII + CD206-, green = CD206 +). Cell numbers for each time point in (**C**) were compared by unpaired t-test of y = ln(y) transformed data *P < 0.05, **P < 0.01, ***P < 0.001, ****P < 0.0001.

As our first in vivo results illustrated only a snapshot of a dynamically regulated system we sought to compare MNP composition between IRI (Fig. 5) and UUO (Fig. 6) in a time-dependent manner over a 10 day time window. IRI kidneys exhibited a robust response in CD86 + MHCII + CD206- cells already one day after injury, that consisted mostly of MNPs from subset 1 and 2 (Fig. 5A,B). The same subsets contributed to CD86 + MHCII + CD206- cells in UUO kidneys, but they peaked only at day 3 after UUO and were preceded by an acute surge of cells from subset 3 (Fig. 6). A continuously mounting secondary response of CD86 + MHCII + CD206- cells from subset 4 was observed in IRI, whereas these cells were already declining after 3 days in UUO kidneys. A statistical comparison between all five MNP subsets and each time point is given in Supplementary Table S1. Interestingly, MNP subset 1 gradually transformed away from an CD86 + MHCII + CD206- into an CD206 + phenotype over time (Figs. 5C and 6C). CD206 + cells in both models peaked after three days and were mostly supported by subset 3 and in some degree by subset 1 and 2. In summary, the UUO model seemed to have a more delayed response in CD86 + MHCII + CD206- cells that involved subset 3 in the acute phase, whereas in contrast, the IRI model had a gradually increasing involvement of subset 4 in this functional subset.

### Ex vivo phagocytosis indicates functional differences between identified subsets

**Figure 7:**
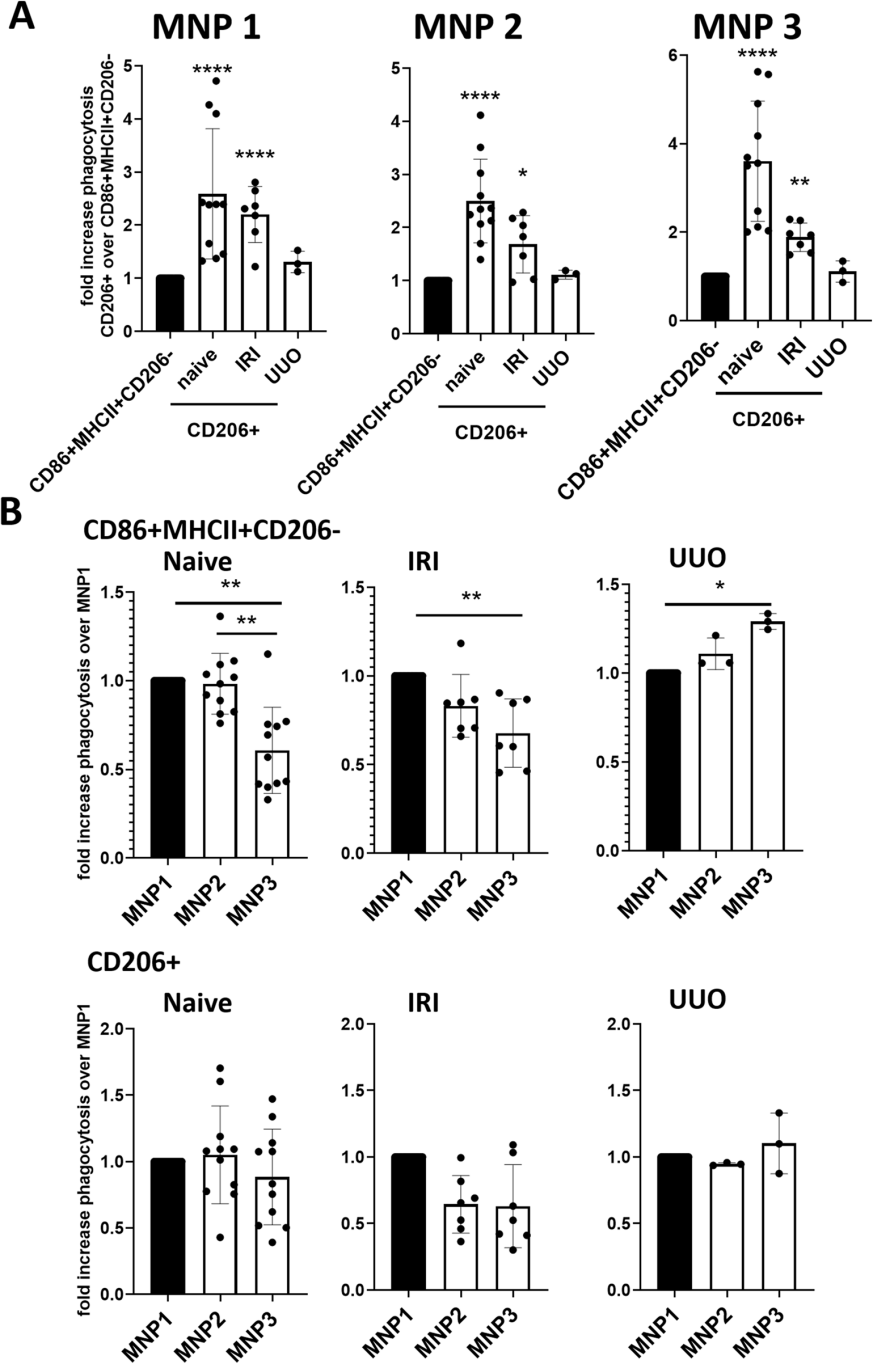
Ex vivo phagocytic capacity is variable between MNP subsets in different kidney injury models. CD11b + CD11c + -sorted cells from kidneys from naïve C57BL6/J (n = 11), 24 h after IRI (n = 7) and 3 days after UUO (n = 3) were fed PE + latex-beads for 2 h and phagocytic cells were identified as PE + events. Phagocytic cells were further dissected with our gating strategy for MNP subsets and CD206 + and CD86 + MHCII + CD206- cells. The data of each replicate were plotted either as (**A**) relative change compared to CD86 + MHCII + CD206- cells [%phagocytic cells/%phagocytic cells in CD86 + MHCII + CD206-] or (**B**) as relative change compared to MNP subset 1 [%phagocytic cells/%phagocytic cells in MNP1]. Data from two to three independent experiments are shown. Kruskal–Wallis test followed by Dunn’s multiple comparisons tests against M1 (**A**) or Friedman with post-hoc Dunn’s multiple comparisons (**B**) *P < 0.05, **P < 0.01, ***P < 0.001, ****P < 0.0001.

In order to explore if CD86 + MHCII + CD206- and CD206 + cells were functionally distinct between different MNP subsets, we assessed their phagocytic capacities in each subset. We therefore quantified the uptake of fluorescently labeled latex beads by sorted MNPs from kidneys from naïve C57BL/J mice, 24 h after IRI, and 3 days after UUO ex vivo (gating strategy is available in Supplementary Figure S6 A). In order to introduce the fluorescent beads into our panel, we omitted Ly6G for this experiment, which did not affect CD86 + MHCII + CD206- and CD206 + cell readouts (Supplementary Figure S6 B). As CD206 + cells in MNP subsets 4 and 5 were not sufficiently present we omitted these subsets for the analysis (Figs. 5 and 6). The phagocytic capacity of CD206 + cells in MNP subsets 1 to 3 was significantly more pronounced than in their CD86 + MHCII + CD206- counterparts in all models except UUO (Fig. 7B). Among the three analyzed MNP subsets, subset 1 had higher phagocytic capacity than subset 3 in the CD86 + MHCII + CD206- cell compartment in UUO, while this observation was reversed for naïve and IRI kidneys (Fig. 7B). These results indicate functional differences between CD86 + MHCII + CD206- cells in each MNP subset.

## Discussion

Here we introduce a flow cytometric method that further dissects phenotypic MNP subsets in the kidney into CD86 + MHCII + CD206- and CD206 + subunits. We incorporated an approach used by Kawakami et al.^17^ to distinguish five renal MNP subsets, which have been demonstrated to possess distinct surface marker expression and phagocytic and antigen-presenting capacities^17,18,38^. We confirmed the confined expression of some of these surface markers and found similar distribution of MNP subsets in naïve kidneys, demonstrating the high reproducibility of this gating approach. In contrast to the original method introduced by Kawakami et al.^17^, we dissected MNP subset 3 before gating the remaining four subsets in order to have better contrast on the CD11b expressing populations. This gate should contain most of the kidney-resident macrophages, as they are F4/80^high^, and should contain none to only little contaminations from monocyte-derived macrophages, as we do not see any Ly6C expression, which is restricted to these cells according to Puranik et al.^12^. Our data demonstrate that all five subsets expand in kidney injury models of IRI, UUO and Alport syndrome, something that is reminiscent of a model of systemic lupus erythematosus^18^. This implicates that all subsets contribute in some ways to inflammation and/or repair during sterile inflammation. Systemic inflammation via injection of LPS on the other hand leads to predominant extravasation of subsets 1 and 3 to draining lymph nodes after 48 h, but an increase in subset 2 in the kidney^17^, suggesting different roles after infectious diseases.

The functional relevance of individual subsets has been a matter of debate and single-cell analyses have made it apparent that MNP subsets may consist both of pro- and anti-inflammatory cells^13,18,19,39^. We implemented a gating strategy, that distinguishes CD86 + MHCII + CD206- and CD206 + cells, on top of the gating for the five MNP subsets in order to create a tool that dissects this next level of functional complexity. Our gating strategy involved MHCII, CD86 and CD206, markers which have already been associated with functionally distinct subsets in murine kidneys^14,34,40,41^. For instance, CD206 + kidney cells were a major source of TGF-β production and fibrotic processes after IRI^36^. On the other hand, transfusion of CD206 + cells has been associated with ameliorated production of inflammatory cytokines and histological injury during nephrotoxic serum-induced glomerulonephritis^29^ or adriamycin-induced nephropathy^28^, while transfusion of CD86 + cells worsened these readouts, respectively.

Although, CD86 + MHCII + CD206- cells might be reminiscent of M1-like cells, while CD206 + cells may resemble M2-like cell, it should be noted that M1/M2 markers can co-exist on individual cells and thus the use of “specific” markers for M1/M2 in vivo is problematic. Along these lines, we note that in our in vivo experiments most CD206 + cells also expressed CD86. Co-expression of these M1- and M2-markers is also seen in other settings^42,43^ and highly context-dependent^32^. Hence, any M1/M2 annotations should be omitted for our in vivo setting.

Analysis of three different models of kidney injury confirmed that basically all five MNP subsets were contributing to both CD86 + MHCII + CD206- and CD206 + cells. Similarly, multimodal expression of pro- and anti-inflammatory cytokines and chemokines among individual MNP subsets has already been observed in single-cell gene expression analyses from mice with rhabdomyolysis-Induced kidney injury^13^ or systemic lupus erythematosus^18,39^. Thus, our results indicate that previous flow cytometry data, that were focused on individual MNP subsets, analyzed a mix of functionally heterogeneous cells^12,16,20^. Inversion of this argument implicates that cells with similar function in the kidney are also heterogenous in their origin of MNP subset and that restrictive analysis of only F4/80^high^ cells disregards CD86 + MHCII + CD206- and CD206 + cells from all other MNP subsets^33,34,36,44,45^. This commonly overlooked functional heterogeneity among cell subsets could also be part of the reason why mechanistic studies depleting macrophages before or after kidney injury have had such vastly different outcomes in regard to kidney function and recovery^21,22^. Cell removal by clodronate liposomes and diphteria toxin may have different impacts on MNP subsets and cannot fully discriminate their function, resulting in high heterogeneity of remaining cells.

We also quantified MNPs in 7 weeks old *Col4a3*^*−/−*^ mice and found more cells across all subsets compared to IRI or UUO. Such dramatic increases in MNP numbers have been observed before in murine models of Alport^46^, but have not been put into perspective to other models of renal disease so far. These comparisons could for example help to explain why removal of 70% of renal macrophages by clodronate liposome treatment in *Col4a3*^*−/−*^ mice did not improve renal function^47^: According to our data 30% remaining MNPs would result in about the same cell burden as in the IRI or UUO model, and again the cell composition of the remaining cells is unknown. Another feature of the Alport model is the very large proportion of MNP3 cells among CD86 + MHCII + CD206- cells, especially when comparing to UUO, which has only few of these cells. These F4/80 + CD206- cells are not efficient TGF-β producers^36^ and thus probably do not contribute to fibrosis as much as their CD206 + counterparts, maybe even having counteracting properties. This might also contribute to the reno-protective effects of F4/80 + cell depletion in UUO^48^, which could not be observed in the Alport model^47^.

Because MNP subsets are dynamically regulated after kidney injury, we performed a longitudinal comparison between two models of kidney injury with our new method. While overall MNP dynamics after IRI and UUO were comparable to previous reports^11,49^, we revealed a rather conserved response of CD206 + cells dominated by subset 3 (F4/80^high^) in both models and responses in CD86 + MHCII + CD206- cells, which were highly model-dependent. Interestingly, CD86 + MHCII + CD206- cells in IRI accumulated over time in MNP subsets 3 and 4. These subsets are reminiscent of Ly6C-F4/80 + cells and CD103 + cells, which are implicated in exacerbated kidney injury and fibrosis by activating myofibroblasts^20^ and CD8^+^ T cells^50^, respectively. On the other side of the coin, F4/80 + cells (MNP subset 3) have also been implicated in tubular repair mechanisms^15^, which might be covered by the CD206 + portion of this subset. The strong contribution of subset 1 and 2 to CD86 + MHCII + CD206- cells goes in line with an overall more inflammatory phenotype of these subsets compared to subset 3 as established by gene expression analysis^11,12^. However, our method also identifies parts of these subsets contributing to CD206 + cells. Indeed, MNP subset 1 gradually switches its phenotype from CD86 + MHCII + CD206- to CD206 + in both IRI and UUO, which argues for a more pro-fibrotic role of these cells in later stages after IRI and UUO. Conditional ablation of CD11b + cells between days 7 to 10 after UUO, which is around the time of the phenotype switch, did indeed reduce fibrosis, while adoptive transfer of monocytes restored fibrosis^49^. In terms of mechanistic considerations our results suggest acute infiltration of proinflammatory monocytes (CD86 + MHCII + CD206- cells in MNP subset 2) after IRI and growing activation of adaptive immunity with large numbers of CD86 + MHCII + CD206- cells in MNP subsets 1, 4 and 5, which are all implicated in antigen-presentation^17,18,51^. CD86 + MHCII + CD206- cells in UUO on the other hand are not accumulating as acute as in IRI and are dominated by high proportions of MNP subset 2 (Ly6C^high^), arguing for persistent monocyte influx from the periphery throughout disease progression and minor contribution of adaptive immune activation. Interestingly, UUO has been associated with persistent infiltration of Ly6C^high^ cells and concomitant inflammation and fibrosis^49,52^. CD11c + cells in MNP subsets 1 and 4, which share resemblance with dendritic cells, are slowly decreasing after day 3 after UUO, which seem in line with a minor contribution to fibrosis in the UUO model despite increased immune activation at that time point^53^. The same subsets seem to have more relevant roles in experimental glomerulonephritis where CD103 + cells attract neutrophils to the kidney while CD11b + CD11c + cells oppose this effect^54^. It would be interesting to see how these functional subsets distribute across our CD86 + MHCII + CD206- and CD206 + subsets in future investigations. Collectively, these results indicate that our flow cytometric approach might delineate novel functional subunits for therapeutic targeting.

One important regulatory function of MNPs after kidney injury is the clearance of apoptotic cells and debris^21,55,56^. The CD206 + cells in subsets 1 to 3 displayed higher phagocytic capacity than their CD86 + MHCII + CD206- counterparts in naïve kidneys and after IRI, confirming their functional distinctness in individual MNP subsets. To our knowledge, this is the first direct ex vivo comparison of phagocytic activity in functional subsets of renal MNPs from IRI and UUO kidneys. The fact that not only the MNP phenotype but also its functional background has an effect on its phagocytic capacity indicates very complex implications and involvement in kidney injury and regeneration. In this regard it is interesting that other studies have suggested that phagocytosis of apoptotic cells by CD301 + MNP (which are also CD206 +) is more pronounced only in later stages after UUO^45^. As all MNP subsets show substantial phagocytic capacity in the naïve kidney^17^, the functional state of MNP subsets seems to be highly dependent on the inflammatory context as also indicated by the differences we observed between naïve, IRI and UUO kidneys and thus, requires further investigation.

Considering the functional complexity of the different MNPs, our flow cytometric approach has a more granular resolution than previous flow data that were focused only on MNP subsets^11,12,16,20,57^ or functional distinction^33,35,36,44^. It also puts RNA data from bulk kidney and even sorted renal macrophages into perspective as they cannot discriminate surface markers indicative of different function on single-cell level^27^. Another benefit compared to RNA-based technologies is the possibility of cell-sorting subpopulations for functional downstream applications. A limitation of our flow cytometric approach is that our dump gate only excludes neutrophils via Ly6G from total leukocytes. Thus, although NK cells may solely provide 3,5% of total CD45 + cells in naïve kidneys^58^ and are mostly described as MHCII negative^59,60^ these cells as well as lymphocytes, albeit being mostly CD11b^−^, may still constitute a small portion of our MNP subset analysis. Inclusion of markers like NK1.1, CD19 or CD326 into a lineage cocktail could help to improve the quality of future experiments. Another limitation is the use of only a restricted number of fluorophores whereas single-cell RNA analysis can deliver thousands of datapoints per cell. Nevertheless, single-cell RNA sequencing of myeloid cells from UUO kidneys identified several heterogenous clusters^61^, some of which may correspond with our flow cytometry approach (such as Ly6C^high^ monocytes as MNP2 and F4/80 + resident macrophages as MNP3) and warrant further investigation as to how CD86 + MHCII + CD206- and CD206 + cells may correspond to other clusters of this study. It is also unclear how these functional subunits translate into the human kidney, although there are correlates to each of the five MNP subsets^51^. Moreover, although one might correlate CD86 + MHCII + CD206- and CD206 + cells to cells with similar surface marker expression from literature, it has to be noted that our MNP subset analysis could not identify direct functional relevance of individual subsets in in vivo models due to lack of appropriate depletion models for this combination of markers. More extensive analysis of MNP subsets at later time points after IRI and UUO that reflect a more chronic phenotype but also other kidney disease models involving diabetes, hypertension or glomerulonephritis might be of interest for future investigations.

In conclusion, our newly established flow cytometric approach offers a tool that dissects the functional heterogeneity of kidney MNP subsets, which has often been disregarded in the past. We also demonstrate that these functionally distinct subsets are dynamically regulated in a manner highly dependent on the context of kidney injury. Dissecting renal MNP on a functional level could give new mechanistic disease understanding and open an avenue for new therapeutic targets in the future.

## Methods

### Animal models

Alport syndrome model: Heterozygous *Col4a3*^+*/−*^ transgenic mice (Jackson Laboratories, USA), backcrossed twice on a 129S2/SvPasCrl genetic background, were crossbred. Genotyping of the resulting 129-*Col4a3*^*tm1Dec*^/J mice (F1) was carried out by polymerase chain reaction (PCR) and kidneys of 7 weeks old homozygous *Col4a3*^*−/−*^ knock-out mice or *Col4a3*^+*/*+^ littermates (naïve) were used for analysis.

Kidney ischemia/reperfusion injury (IRI) model: C57BL/6 male mice were obtained from Charles River (Sulzfeld, Germany). At the age of 8 weeks mice were anesthetized and subjected to renal IRI by clamping the left renal pedicle for 25 min using a nontraumatic micro-aneurysm clip (Thermo Fisher Scientific, Waltham, MA). Mice were kept at 37 °C using a warming pad and reperfusion of the left kidney was confirmed after clamp release. Alternatively, “sham” surgery (flank incision only) was performed on age-matched male C57BL/6 mice. Mice were given 1 ml of normal saline intraperitoneally to prevent dehydration.

Kidney unilateral ureteral obstruction (UUO) model: For UUO 8 weeks old male C57BL/6 mice were anesthetized and the left ureter was ligated twice with 5/0 Seraflex silk suture (Serag-Wiessner, Neila, Germany) and afterwards the ureter was cut between the sutures. After 3 h, 1 day, 3 days, 7 days and 10 days mice were sacrificed, and the kidneys were removed for downstream applications. Alternatively, “sham” surgery (flank incision only) was performed on age-matched male C57BL/6 mice.

All procedures conformed with European (Guideline 2010/63/EU) and national legislation (dt. Tierschutzgesetz v. 04.07.2013) for the use and protection of animals for scientific purposes. Experiments were approved by the institutional animal care office of Bayer AG and by the competent regional authority (*Landesamt für Natur, Umwelt und Verbraucherschutz Nordrhein-Westfalen (LANUV))* which has an ethics committee. All methods were performed in accordance with the ARRIVE guideline and other relevant guidelines and regulations.

### Flow cytometry

For flow cytometry, single-cell suspensions of the kidney were made by digesting kidney tissue with the Multi Tissue Dissciation Kit 1 (Miltenyi Biotec, Bergisch Gladbach, Germany) according to the user manual. Following filtration through a 70 µM mesh, erythrocytes were lysed using BD Pharm Lyse (BD Biosciences) and washed with autoMACS Running Buffer (Miltenyi Biotec). Fc receptors were blocked with CD16/CD32 (BD Biosciences) for 10 min and extracellular surface markers were stained with an antibody cocktail containing FITC anti-mouse CD86 (clone GL-1, BioLegend, San Diego, CA), BV51 anti-mouse CX3CR1 (clone SA011F11, BioLegend), PE anti-mouse F4/80 (clone T45-2342, BD Biosciences, FranklinLakes, NJ), PerCP-Cy5.5 anti-mouse CD11b (clone M1/70, BD), PE-Cy7 anti-mouse CD11c (clone HL3, BD), APC anti-mouse Ly-6G (clone 1A8, BD), APC-Cy7 anti-mouse CD45 (clone 30-F11, BD), V500 anti-mouse I-A/I-E (clone M5/114.15.2, BD), FITC anti-mouse Ly-6C (clone AL-21, BD), FITC anti-mouse CD103 (clone M290, BD), FITC Rat IgG2a, κ isotype control (clone R35-95, BD), PE-Cy7 Hamster IgG1, λ1 isotype control (clone G235-2356, BD), BV421 Rat IgG2a, κ isotype control (clone R35-95, BD) each at a 1:100 dilution. For intracellular CD206 staining, cells were fixated for 10 min with Leucoperm reagent A and after a washing step permeabilized with Leucoperm reagent B (Bio-Rad, Hercules, CA) containing 1:100 BV421 anti-mouse CD206 (clone C068C2, BioLegend). After washing, cells were analyzed on a BD FACSVerse Flow Cytometer (BD Biosciences). Data analysis and graph generation was performed using FlowJo 7.6 software (TreeStar, Ashland, OR).

### Macrophage isolation and polarization

Bone marrow-derived macrophages (BMDM) were acquired by flushing out bone marrow by gravitational force in a microcentrifuge from femurs and tibia. Following erythrocyte lysis using BD Pharm Lyse (BD Biosciences), cells were filtered through a 70 µM mash and washed with Dulbecco’s modified Eagle’s medium (DMEM) HAM’s F12 (Thermo Fisher Scientific) supplemented with 10%FCS and 1% penicillin–streptomycin (both Thermo Fisher Scientific). 3 × 10e5 cells were seeded into Corning CellBIND 12-well plates (Corning, New York, NY) in full DMEM supplemented with 20 ng/ml macrophage colony-stimulating factor (M-CSF; Peprotech, London, UK) and cultured at 5% CO_2_ at 37 °C. After 3 days half of the medium was refreshed and on day 5 medium was replaced with full DMEM for 48 h to obtain M0, stimulated with lipopolysaccharide (LPS; 2,5 µg/ml, Sigma-Aldrich, St. Louis, MO) for 2 h to obtain M1, or stimulated with IL-4 and IL-13 (both 10 ng/ml, Thermo Fisher Scientific) for 48 h to obtain M2. For qPCR analysis cell were washed in PBS and dissolved in RLT buffer (Qiagen, Hilden, Germany). Cells for flow cytometry were scraped, washed in PBS and submitted to FACS staining as described above.

### Ex vivo phagocytosis assay

Kidneys from naïve C57Bl/6 mice, after 24 h IRI and 3 days UUO were digested as described above and renal MNPs were sorted using CD11b- and CD11c-MicroBeads on LD columns (both Miltenyi Biotec) according to the instruction manual. Phagocytic capacity was assessed using the Phagocytosis Assay Kit (IgG PE) (Cayman Chemical, Ann Arbor, MI) according to the instruction manual. Briefly, sorted cells were incubated in RPMI containing PE-labeled latex beads for 2 h at 37 °C and washed in assay buffer. In the FACS panel for the phagocytosis assay APC-Ly-6G and BV421-CD206 were replaced by BV421 Rat Anti-Mouse F4/80 (clone T45-2342, BD) and APC anti-mouse CD206 (clone C068C2, BioLegend). Staining, including intracellular staining of CD206, were performed as described in the flow cytometry methods. The percentage of PE + phagocytic cells was determined for each MNP subset and the relative change between subsets was calculated for each replicate.

### Real-time PCR analysis

RNA was extracted with a RNeasy Mini kit (Qiagen) and submitted to reverse transcription. Gene expression analysis was determined by quantitative real-time Taqman PCR using an ABI 7900HT Fast Real Time PCR System (Thermo Fisher Scientific) and normalized to RPL32 as housekeeper, which shows in our experience least regulation in various in vitro and in vivo models of the cardiovascular indication. The following primers and FAM/TAMRA-labelled probes were used: RPL32 Fw: ACCGAAAAGCCATTGTAGAAAGA; Rev: CCTGGCGTTGGGATTGG; probe: CAGCACAGCTGGCCATC; CD86 Fw: GAGTTTCCATCTGCTCAAACG; Rev: ACTTAGAGGCTGTGTTGCTG; probe: CCTGCTAGGCTGATTCGGCTTCT; SOCS3 Fw: GAAGATTCCGCTGGTACTGAG; Rev: GCTGGGTCACTTTCTCATAGG; probe: CCGACAAAGATGCTGGAGGGTGG; TNFα Fw: CTTCTGTCTACTGAACTTCGGG; Rev: CAGGCTTGTCACTCGAATTTTG; probe: ATCTGAGTGTGAGGGTCTGGGC; Mrc1 Fw: ATGGATGTTGATGGCTACTGG; Rev: TTCTGACTCTGGACACTTGC; probe: ACGAAATCCCTGCTACTGAACCTCC; CD200r1 Fw: GAGAAAAGGTACCGAGTGAGC; Rev: ATCAGTACAACTTGACCCAGC; probe: TGTTTTGCTTTTGGAGAACTTCTGCCC. Gene expression was normalized to RPL32 as housekeeping gene. Data are expressed using the 2-ΔΔCTmethod, and mRNA expression is are calculated as fold over basal.

### Statistics

All statistics and respective graphs were generated using GraphPad Prism 8.0.2 for Windows (GraphPad Software, San Diego, CA) and data presented as Mean ± S.E.M. In order to achieve normal distribution of MNP cell numbers, data were y = ln(y) transformed before analysis. Multiple groups were tested for significance using one-way ANOVA (normal data) or Kruskal–Wallis (non-normal data) followed by Tukey’s, Dunnett’s or Dunn’s multiple comparisons test as indicated in figure legends. MNP subsets at different time points of the IRI and UUO model were compared using two-way ANOVA with Tukey’s multiple comparisons test. For paired tests in the ex vivo phagocytosis assay we used Friedman with post-hoc Dunn’s multiple comparisons for multiple data sets.

## Supplementary Information

Supplementary Information.

## Data Availability

The datasets generated during and/or analysed during the current study are available from the corresponding author on reasonable request.
