# Supplementary material for: A flow cytometry approach reveals heterogeneity in conventional subsets of murine renal mononuclear phagocytes
Source: Sci Rep. 2021 Jun 24;11:13251. doi: 10.1038/s41598-021-92784-x (PMC8225656; doi:10.1038/s41598-021-92784-x)
Supplement: Supplementary file 1 — Supplementary Information. [file 41598_2021_92784_MOESM1_ESM.pdf]

- Supplementary Information -

A flow cytometry approach reveals heterogeneity in conventional subsets of murine renal mononuclear phagocytes

*Johannes Nordlohne[1], Ilona Hulsmann[1], Svenja Schwafertz[1], Jasmin Zgrajek[1], Manuel Grundmann[1], Sibylle von Vietinghoff[2], Frank Eitner[1], Michael S. Becker[1]**

[1]Cardiovascular Research, Bayer AG, Pharma Research Center, Wuppertal, Germany

[2]Nephrology Section, Medical Clinic 1, University Hospital Bonn, Rheinische Friedrich-Wilhelms University, Bonn

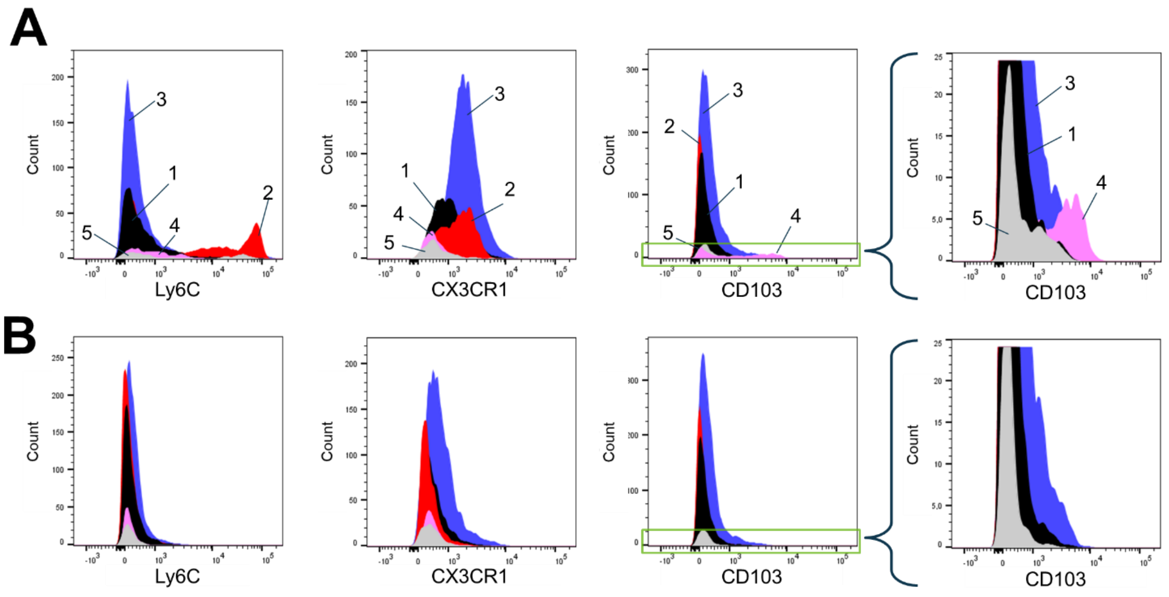

Supplementary Figure S1: **FMO controls for *in vivo* surface marker expression.** Histograms for surface marker expression of Ly6C, CX3CR1 and CD103 assessed by flow cytometry on MNP subsets 1-5 in digested kidneys (A) and their respective FMO controls (B).

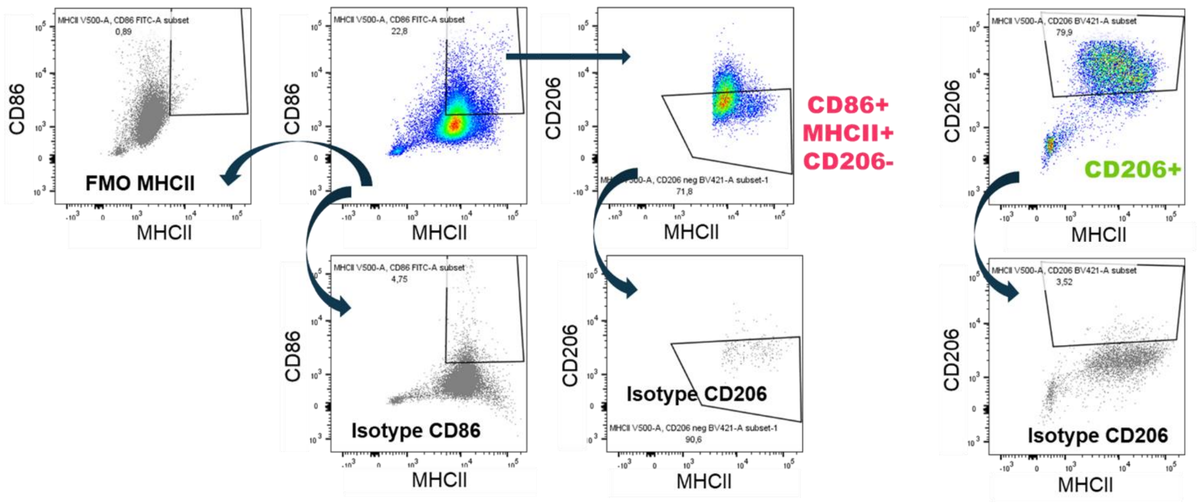

Supplementary Figure S2: **FMO and isotype controls for *in vitro* CD86+MHCII+CD206- and CD206+ cells gating.** *In vitro* polarized BMDM were submitted to flow cytometry and pre-gated on live CD45+ cells. FMO control for MHCII and isotype controls for CD86 and CD206 are depicted.

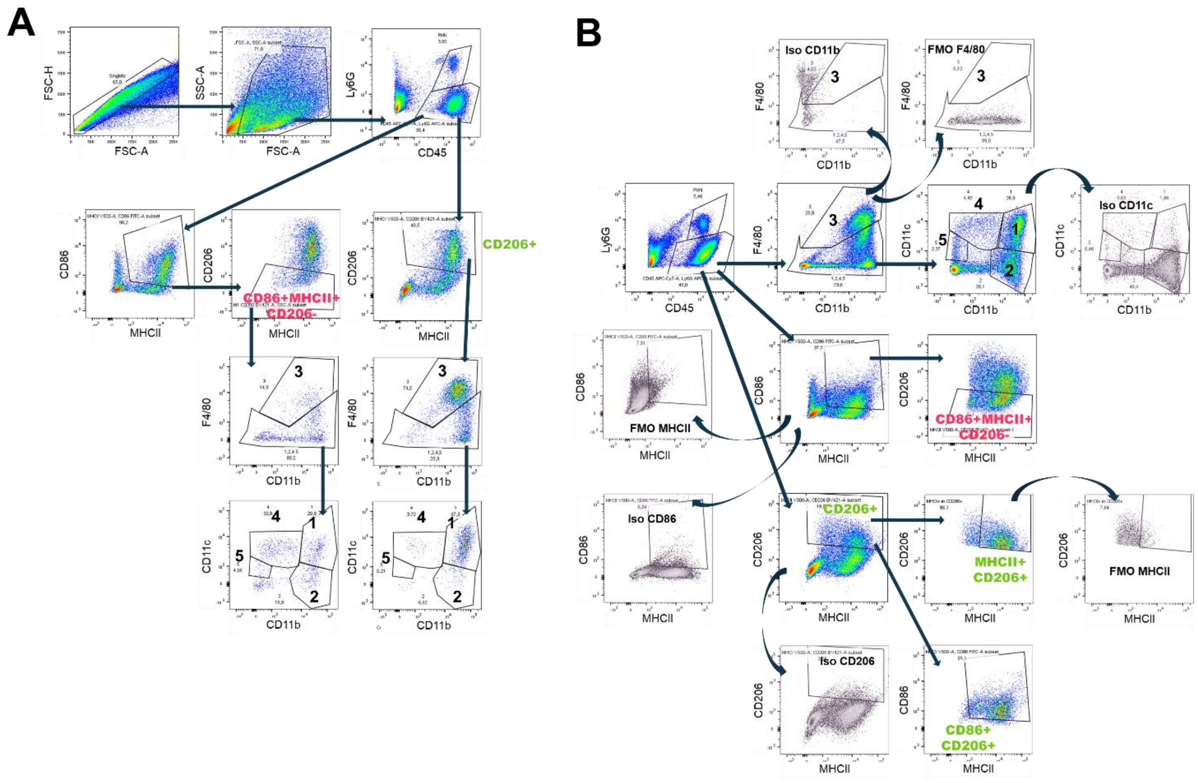

Supplementary Figure S3: **Combined gating strategy for CD86+MHCII+CD206- and CD206+ cells and five MNP subsets with FMO and isotype controls.** (A) Representative FACS plots of a digested kidney 7 days after IRI are shown. After exclusion of duplets and granulocytes, we performed gating for CD86+MHCII+CD206- and CD206+ cells. We then followed up both paths with the gating strategy by Kawakami et. al, which distinguishes five MNP subsets as indicated. (B) FMO control for MHCII and isotype controls for F4/80, CD11b, CD11c, CD86 and CD206 are depicted in relevant leukocyte populations from digested kidneys.

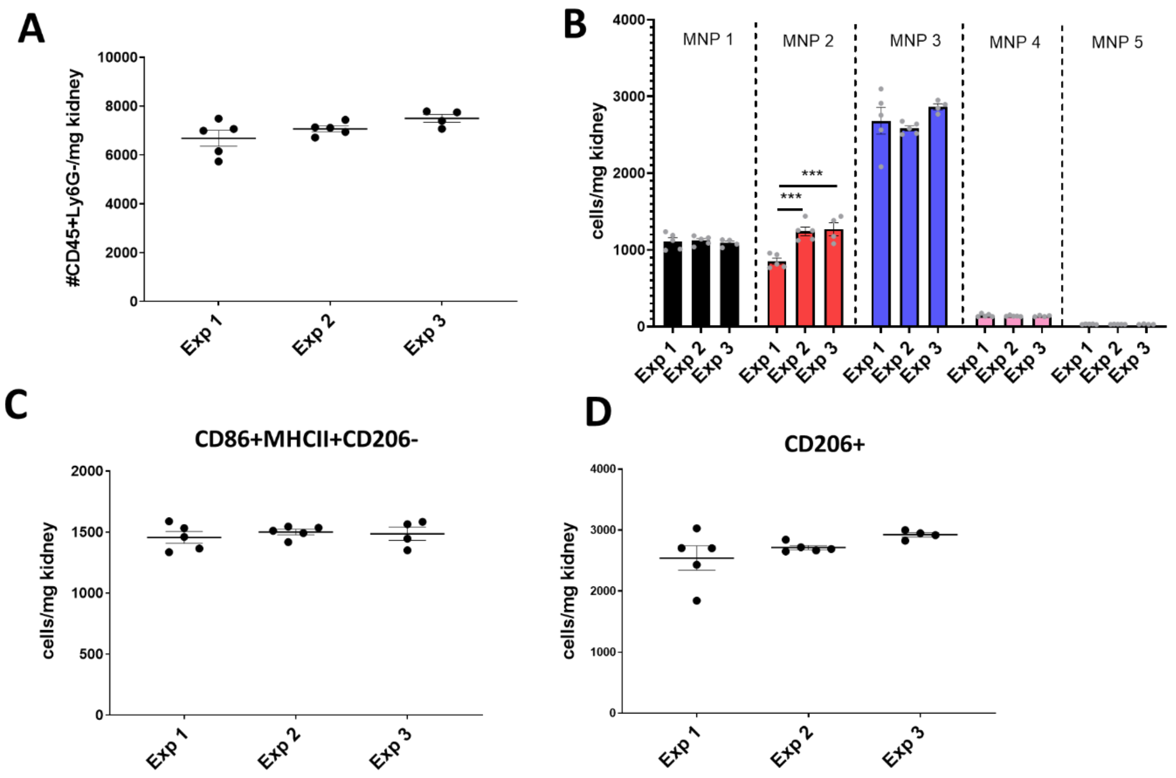

Supplementary Figure S4: **Technical replicates validate robustness of quantification of MNP subsets with flow cytometry.** Kidney homogenate was evenly distributed among three individual experimenters (Exp) and staining protocol and FACS were performed in triplicates. Analysis with identical gating revealed very low standard deviation among triplicates and experimenters for (A) total CD45+Ly6G- leukocytes, (B) MNP subsets and (C) CD86+MHCII+CD206- and (D) CD206+ cells. One-way ANOVA followed by Tukey´s multiple comparisons test of y=ln(y) transformed data, *P<0.05, **P<0.01, ***P<0.001, ****P<0.0001.

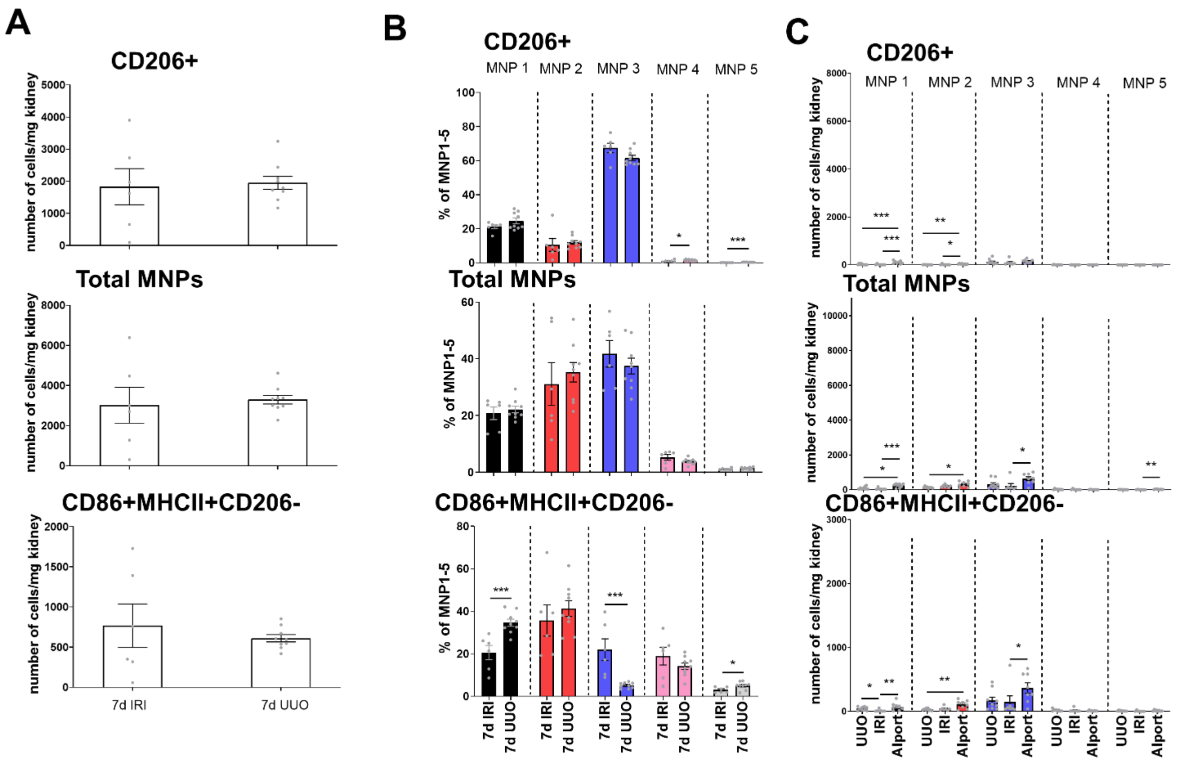

Supplementary Figure S5: **CD206+ and CD86+MHCII+CD206- cells in MNP subsets have different abundances in IRI and UUO.** (A) Cumulative cell number of MNP subets 1-5 given as cells per mg kidney in IRI (n=6) and UUO (n=9) (corresponds to pie chart size in Figure 4A). (B) Proportion of each MNP subset among the cumulative amount of MNPs in IRI and UUO. Mann-Whitney test, *P<0.05, **P<0.01, ***P<0.001, ****P<0.0001. (C) Comparison of MNP distribution in “healthy” control kidney from different kidney injury models: “sham” kidneys from mice 7 days after IRI or UUO and naïve kidneys from Col4a3+/+ mice (Alport model). One-way ANOVA followed by Tukey´s multiple comparisons test of y=ln(y) transformed data, *P<0.05, **P<0.01, ***P<0.001, ****P<0.0001.

Supplementary Table S1 **Statistical analysis of MNP subsets 1-5 at different time points after IRI or UUO from Figure 5B and 6B.**

**Total MNPs**		0 hours	3 hours	1 day	3 days	7 days	10 days
IRI	MNP1 vs. MNP2	ns	****	ns	ns	ns	ns
	MNP1 vs. MNP3	*	*	ns	ns	ns	ns
	MNP1 vs. MNP4	**	**	****	****	ns	****
	MNP1 vs. MNP5	****	*	****	****	**	****
	MNP2 vs. MNP3	ns	*	***	ns	ns	***
	MNP2 vs. MNP4	****	****	****	****	ns	*
	MNP2 vs. MNP5	****	****	****	****	**	****
	MNP3 vs. MNP4	****	****	****	****	ns	****
	MNP3 vs. MNP5	****	****	****	****	***	****
	MNP4 vs. MNP5	ns	ns	**	***	ns	****
UUO	MNP1 vs. MNP2	ns	ns	*	ns	*	ns
	MNP1 vs. MNP3	*	*	ns	ns	*	*
	MNP1 vs. MNP4	**	***	****	****	****	****
	MNP1 vs. MNP5	****	****	****	****	****	****
	MNP2 vs. MNP3	ns	ns	ns	ns	ns	ns
	MNP2 vs. MNP4	****	****	****	****	****	****
	MNP2 vs. MNP5	****	****	****	****	****	****
	MNP3 vs. MNP4	****	****	****	****	****	****
	MNP3 vs. MNP5	****	****	****	****	****	****
	MNP4 vs. MNP5	ns	ns	ns	***	****	**

**CD86+MHCII+CD206-**		0 hours	3 hours	1 day	3 days	7 days	10 days
IRI	MNP1 vs. MNP2	ns	*	ns	ns	ns	ns
	MNP1 vs. MNP3	*	ns	**	ns	ns	ns
	MNP1 vs. MNP4	ns	*	****	**	ns	ns
	MNP1 vs. MNP5	**	*	****	****	ns	****
	MNP2 vs. MNP3	**	ns	****	ns	ns	ns
	MNP2 vs. MNP4	ns	****	****	**	ns	ns
	MNP2 vs. MNP5	**	****	****	****	*	****
	MNP3 vs. MNP4	****	****	**	ns	ns	ns
	MNP3 vs. MNP5	****	****	****	****	ns	***
	MNP4 vs. MNP5	ns	ns	**	****	ns	****
UUO	MNP1 vs. MNP2	ns	ns	ns	ns	ns	ns
	MNP1 vs. MNP3	*	*	ns	****	****	****
	MNP1 vs. MNP4	ns	*	***	****	****	****
	MNP1 vs. MNP5	**	***	****	****	****	****
	MNP2 vs. MNP3	**	**	ns	****	****	****
	MNP2 vs. MNP4	ns	ns	****	****	****	****
	MNP2 vs. MNP5	**	*	****	****	****	****
	MNP3 vs. MNP4	****	****	***	ns	****	ns
	MNP3 vs. MNP5	****	****	****	****	ns	*
	MNP4 vs. MNP5	ns	ns	ns	****	****	**

**CD206+**		0 hours	3 hours	1 day	3 days	7 days	10 days
IRI	MNP1 vs. MNP2	ns	ns	ns	ns	ns	****
	MNP1 vs. MNP3	***	****	ns	*	ns	*
	MNP1 vs. MNP4	**	ns	****	****	**	****
	MNP1 vs. MNP5	***	ns	****	****	****	****
	MNP2 vs. MNP3	****	***	ns	****	ns	****
	MNP2 vs. MNP4	ns	ns	****	****	*	***
	MNP2 vs. MNP5	*	ns	****	****	***	****
	MNP3 vs. MNP4	****	****	****	****	****	****
	MNP3 vs. MNP5	****	**	****	****	****	****
	MNP4 vs. MNP5	ns	ns	*	**	ns	****
UUO	MNP1 vs. MNP2	ns	ns	ns	ns	**	**
	MNP1 vs. MNP3	***	***	*	*	***	**
	MNP1 vs. MNP4	**	***	****	****	****	****
	MNP1 vs. MNP5	***	****	****	****	****	****
	MNP2 vs. MNP3	****	****	ns	*	****	****
	MNP2 vs. MNP4	ns	*	****	****	****	****
	MNP2 vs. MNP5	*	****	****	****	****	****
	MNP3 vs. MNP4	****	****	****	****	****	****
	MNP3 vs. MNP5	****	****	****	****	****	****
	MNP4 vs. MNP5	ns	ns	**	****	****	****

One-way ANOVA with post-hoc Tukey´s multiple comparisons test on y=ln(y) transformed data, ns=not significant, *P<0.05, **P<0.01, ***P<0.001, ****P<0.0001.

**Total MNPs**		MNP1	MNP2	MNP3	MNP4	MNP5
IRI	0 hours vs 3 hours	ns	ns	ns	*	ns
	0 hours vs 1 day	****	****	ns	ns	ns
	0 hours vs 3 days	****	****	****	***	ns
	0 hours vs 7 days	**	*	*	**	ns
	0 hours vs 10 days	****	*	***	****	*
	3 hours vs 1 day	****	*	ns	****	ns
	3 hours vs 3 days	****	ns	****	****	*
	3 hours vs 7 days	****	ns	****	****	*
	3 hours vs 10 days	****	ns	****	****	****
	1 day vs 3 days	ns	ns	***	ns	ns
	1 day vs 7 days	ns	ns	ns	ns	ns
	1 day vs 10 days	ns	ns	**	****	**
	3 day vs 7 days	ns	ns	ns	ns	ns
	3 day vs 10 days	ns	ns	ns	ns	ns
	7 day vs 10 days	ns	ns	ns	ns	ns
UUO	0 hours vs 3 hours	*	ns	*	ns	ns
	0 hours vs 1 day	**	****	ns	ns	ns
	0 hours vs 3 days	****	****	****	****	****
	0 hours vs 7 days	****	****	****	****	****
	0 hours vs 10 days	****	****	**	***	*
	3 hours vs 1 day	ns	***	ns	ns	ns
	3 hours vs 3 days	****	****	**	****	*
	3 hours vs 7 days	****	****	*	****	ns
	3 hours vs 10 days	ns	**	ns	ns	ns
	1 day vs 3 days	****	ns	****	***	***
	1 day vs 7 days	**	ns	****	***	**
	1 day vs 10 days	ns	ns	ns	ns	ns
	3 day vs 7 days	ns	ns	ns	ns	ns
	3 day vs 10 days	**	**	ns	*	ns
	7 day vs 10 days	ns	ns	ns	ns	ns

**CD86+MHCII+CD206-**		MNP1	MNP2	MNP3	MNP4	MNP5
IRI	0 hours vs 3 hours	ns	*	ns	ns	ns
	0 hours vs 1 day	****	****	ns	ns	ns
	0 hours vs 3 days	****	****	ns	***	ns
	0 hours vs 7 days	ns	**	ns	**	ns
	0 hours vs 10 days	**	*	ns	****	*
	3 hours vs 1 day	****	**	ns	**	ns
	3 hours vs 3 days	****	ns	ns	****	ns
	3 hours vs 7 days	*	ns	ns	****	ns
	3 hours vs 10 days	***	ns	ns	****	*
	1 day vs 3 days	ns	ns	ns	ns	ns
	1 day vs 7 days	ns	ns	ns	ns	ns
	1 day vs 10 days	ns	ns	ns	****	*
	3 day vs 7 days	ns	ns	ns	ns	ns
	3 day vs 10 days	ns	ns	ns	ns	*
	7 day vs 10 days	ns	ns	ns	ns	ns
UUO	0 hours vs 3 hours	**	ns	**	ns	*
	0 hours vs 1 day	***	****	ns	ns	ns
	0 hours vs 3 days	****	****	ns	****	****
	0 hours vs 7 days	****	****	***	****	****
	0 hours vs 10 days	**	****	***	*	*
	3 hours vs 1 day	ns	****	*	ns	ns
	3 hours vs 3 days	****	****	**	***	ns
	3 hours vs 7 days	ns	***	****	**	ns
	3 hours vs 10 days	ns	ns	****	ns	ns
	1 day vs 3 days	***	ns	ns	***	***
	1 day vs 7 days	ns	ns	***	***	**
	1 day vs 10 days	ns	*	***	ns	ns
	3 day vs 7 days	*	ns	**	ns	ns
	3 day vs 10 days	****	***	***	*	ns
	7 day vs 10 days	ns	ns	ns	*	ns

**CD206+**		MNP1	MNP2	MNP3	MNP4	MNP5
IRI	0 hours vs 3 hours	**	ns	ns	ns	****
	0 hours vs 1 day	****	****	ns	ns	ns
	0 hours vs 3 days	****	****	****	ns	ns
	0 hours vs 7 days	****	****	***	ns	ns
	0 hours vs 10 days	****	****	****	***	ns
	3 hours vs 1 day	****	****	****	ns	****
	3 hours vs 3 days	****	****	****	ns	****
	3 hours vs 7 days	****	****	****	*	****
	3 hours vs 10 days	****	****	****	***	****
	1 day vs 3 days	ns	ns	***	ns	ns
	1 day vs 7 days	ns	ns	ns	ns	ns
	1 day vs 10 days	ns	ns	***	*	ns
	3 day vs 7 days	ns	ns	ns	ns	ns
	3 day vs 10 days	ns	ns	ns	ns	ns
	7 day vs 10 days	ns	ns	ns	ns	ns
UUO	0 hours vs 3 hours	ns	**	ns	ns	ns
	0 hours vs 1 day	***	****	ns	ns	ns
	0 hours vs 3 days	****	****	****	****	ns
	0 hours vs 7 days	****	****	****	****	**
	0 hours vs 10 days	****	****	****	****	ns
	3 hours vs 1 day	ns	****	ns	ns	ns
	3 hours vs 3 days	****	****	****	***	ns
	3 hours vs 7 days	****	****	****	****	**
	3 hours vs 10 days	****	****	***	**	ns
	1 day vs 3 days	****	****	****	*	ns
	1 day vs 7 days	****	ns	****	***	**
	1 day vs 10 days	***	ns	**	ns	ns
	3 day vs 7 days	ns	*	ns	ns	ns
	3 day vs 10 days	ns	****	ns	ns	ns
	7 day vs 10 days	ns	ns	ns	ns	ns

Two-way ANOVA with post-hoc Tukey´s multiple comparisons test on y=ln(y) transformed data, ns=not significant, *P<0.05, **P<0.01, ***P<0.001, ****P<0.0001.

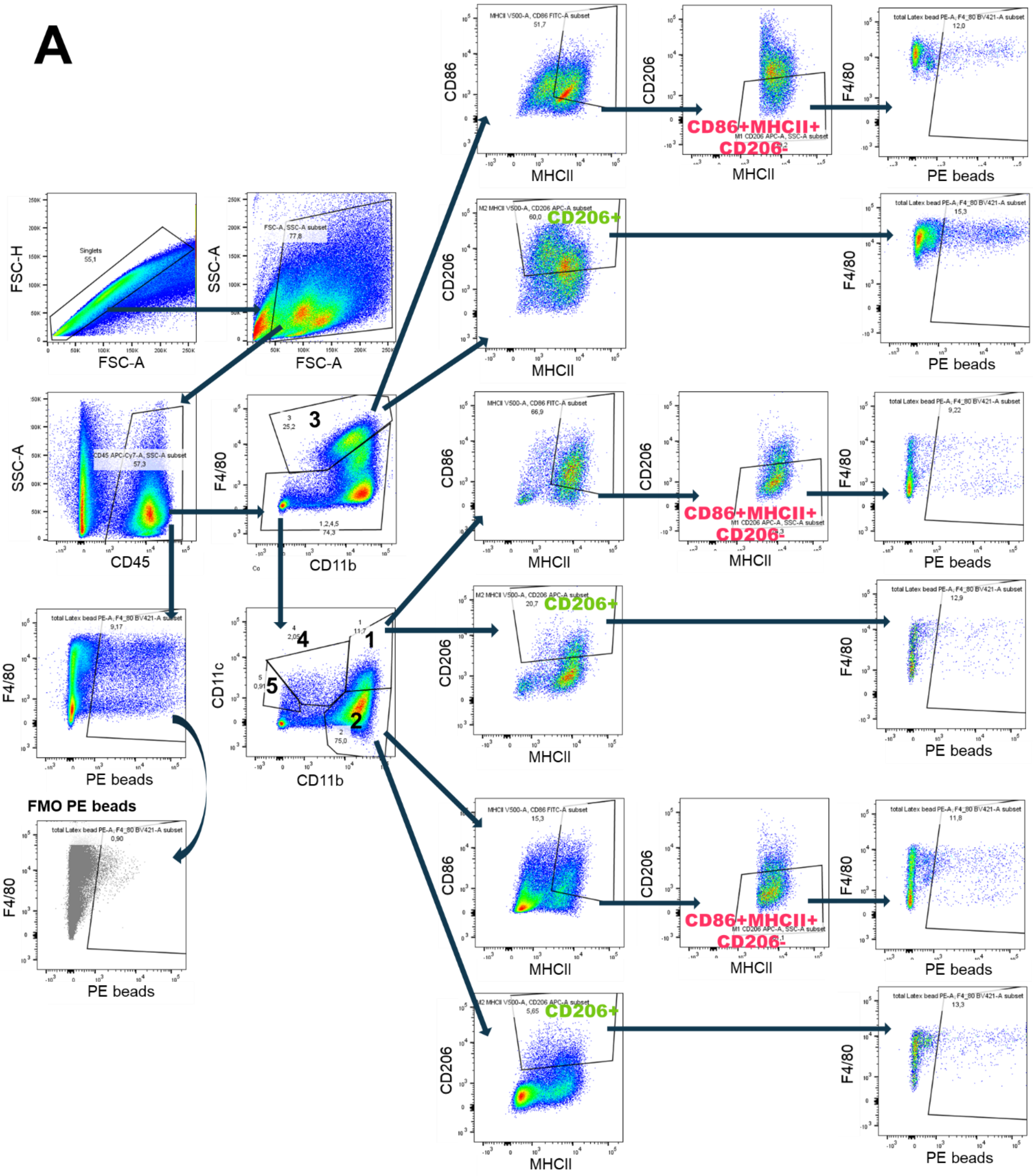
A
FSC-H
FSC-A
Singlets
55,1
SSC-A
FSC-A, SSC-A subset
77,8
CD45 APC-Cy7-A, SSC-A subset
57,3
CD45
F4/80
CD11b
3
25,2
1,2,4,5
74,3
CD11c
4
2,05
1
11,7
5
0,91
2
75,0
total Latex bead PE-A, F4_80 BV421-A subset
9,17
PE beads
FMO PE beads
0,90
CD86
MHCII
MHCII V500-A, CD86 FITC-A subset
51,7
CD206
CD86+MHCII+
CD206-
M1 CD206 APC-A, SSC-A subset
12,0
M2 MHCII V500-A, CD206 APC-A subset
60,0
CD206+
15,3
66,9
9,22
20,7
12,9
15,3
11,8
5,65
13,3
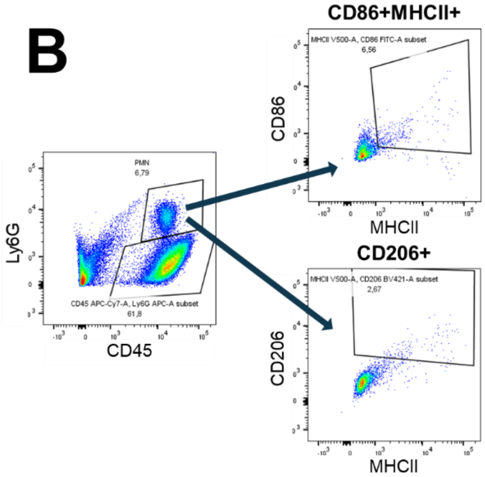
B
Ly6G
CD45
PMN
6,79
CD45 APC-Cy7-A, Ly6G APC-A subset
61,8
CD86+MHCII+
CD86
MHCII
MHCII V500-A, CD86 FITC-A subset
6,56
CD206+
CD206
MHCII V500-A, CD206 BV421-A subset
2,67

Supplementary Figure S6: **Gating strategy for *ex vivo* phagocytosis assay.** (A) MNPs from *ex vivo* phagocytosis assay were pre-gated on CD45+ leukocytes and then submitted to gating for five MNP subsets based on Kawakami et al. followed by gating for CD86+MHCII+CD206- and CD206+ cells for MNP subsets 1-3. The gating was concluded by applying the gate for PE+ phagocytic cells to each of the previous gates. (B) In order to include PE beads into our panel we omitted Ly6G for the *ex vivo* phagocytosis assay. MHCII- and CD206-expression on Ly6G+ cells indicates no contribution of these cells to the CD86+MHCII+CD206- and CD206+ cells.
